# Wild Watermelon-Extracted Juice Ingestion Reduces Peripheral Arterial Stiffness with an Increase in Nitric Oxide Production: A Randomized Crossover Pilot Study

**DOI:** 10.3390/nu14245199

**Published:** 2022-12-07

**Authors:** Shumpei Fujie, Keiko Iemitsu, Kenichiro Inoue, Taro Ogawa, Ayaka Nakashima, Kengo Suzuki, Motoyuki Iemitsu

**Affiliations:** 1 Faculty of Sport and Health Science, Ritsumeikan University, Kusatsu 525-8577, Japan; 2 Research Fellow of the Japan Society for the Promotion of Science, Tokyo 102-0083, Japan; 3 Euglena Co., Ltd., Tokyo 108-0014, Japan

**Keywords:** wild watermelon, arterial stiffness, pulse wave velocity, blood flow, nitric oxide, vasodilation

## Abstract

Wild watermelon contains various nutrients, but the effect of its acute ingestion on arterial stiffness is unclear. This study aimed to investigate whether a single bout of acute ingestion of wild watermelon-extracted juice decreased arterial stiffness concomitant with an increase in nitric oxide (NO) production. Twelve healthy young female participants were tested under two conditions in a randomized, double-blind crossover study: (1) a beverage containing 90 g of wild watermelon extract and (2) a control beverage: a placebo. Pulse wave velocity (PWV), an index of arterial stiffness, blood flow, and plasma nitrate/nitrite (NOx) levels were measured in the supine position at 30, 60, and 90 min after the intake of each beverage. The changes in femoral-ankle PWV were significantly reduced after wild watermelon-extracted juice intake compared to those in the placebo group. Additionally, the changes in blood flow in the posterior tibial artery and plasma NOx levels after intake of wild watermelon-extracted juice were significantly increased compared to those in the placebo group. These data show that acute ingestion of wild watermelon-extracted juice reduces peripheral (lower limb) arterial stiffness and increases NO bioavailability. To confirm these associations, more detailed investigations of the nutrients that influence these effects should be conducted.

## 1. Introduction

Cardiovascular disease is the leading cause of death and arterial stiffness is an independent predictor of cardiovascular disease [1]. It is related to the development of several pathological conditions such as congestive heart failure, atherosclerosis, stroke, and hypertension [2,3]. Vascular endothelial cells regulate vascular tone and structure by producing and releasing nitric oxide (NO), a potent vasodilator [4]. NO, which is produced by NO synthase (NOS) from the amino acid L-arginine, induces a reduction in arterial stiffness [5]. Increased arterial NO production by dietary nitrate [6] and L-citrulline [7] intake and regular aerobic exercise [8] promotes vasodilation, resulting in a reduction in arterial stiffness.

L-citrulline was first extracted from watermelon juice in 1930. Watermelon (*Citrullus lanatus*) is a rich source of L-citrulline [9]. Wild watermelon (*Citrullus lanatus* sp.), which can adapt and grow under high-ultraviolet light and in severely dry conditions, comes from the Kalahari Desert in southern Africa as its place of origin [10]. Wild watermelon is one of the few food items rich in citrulline and arginine [11]. Chronic L-citrulline supplementation induces an increase in nitrate/nitrite (NOx) [7], which are the final metabolites of NO, or artery flow-mediated dilation [12], and the reduction of arterial stiffness [13] in healthy adults. In contrast, an acute combined intake of L-citrulline and L-arginine increased NOx levels in plasma and blood flow in rodents, but ingestion of each alone was ineffective [14]. Thus, supplementation with wild watermelon extract may be a more effective dietary intervention for increasing blood flow and vasodilation via increased NO production. Indeed, chronic watermelon supplementation induces higher plasma NOx levels concomitant with increased plasma levels of L-arginine and L-citrulline in healthy young adults [15]. However, the effects of a single ingestion of wild watermelon-extracted juice on systemic and/or local arterial stiffness and NO production in humans remain unclear.

Therefore, we hypothesized that a single bout of acute ingestion of wild watermelon-extracted juice would decrease arterial stiffness concomitant with an increase in NO production. To test this hypothesis, we examined the effects of acute ingestion of a wild watermelon-extracted beverage on systemic and local arterial stiffness and plasma NOx levels in young female participants in a randomized, double-blind crossover study.

## 2. Materials and Methods

### 2.1. Study Participants

Twelve healthy young female participants (height: 160.0 ± 1.0 cm, body weight: 52.6 ± 1.2 kg, body mass index: 20.5 ± 0.4 kg/m^2^, age: 20.3 ± 0.1 years) volunteered to participate in this study. In this study, there were no subjects who habitually exercised (e.g., aerobic and/or resistance training), had cardiovascular disease, smoked, or used diet supplements, prescriptions, or over-the-counter medications. All subjects in this study provided written informed consent for this study, which was approved by the Ethics Committee of Ritsumeikan University. This study was conducted in accordance with the principles of the Declaration of Helsinki. 

### 2.2. Study Design

Each subject was tested under two conditions in a randomized, double-blind crossover study: (1) a beverage containing 90 g of wild watermelon extract (100 mL; calorie: 8.1 kcal, protein: 0.5 g, carbohydrate: 0.2 g, fat: 0.1 g, natrium: 1.3 mg, citrulline: 162 mg, arginine: 30 mg), (2) a control beverage: a placebo (100 mL, 0 g of wild watermelon extract; calorie: 0 kcal, protein: 0 g, carbohydrate: 0 g, fat: 0 g, natrium: 0 mg, citrulline: 0 mg, arginine: 0 mg). On the day of the experiment, pulse wave velocity (PWV), systolic blood pressure (SBP), diastolic blood pressure (DBP), heart rate (HR), blood flow, and blood sampling were measured in the supine position after at least 15 min of rest (pre) and at 30, 60, and 90 min after each beverage ingestion. Fasting plasma and serum blood samples were drawn after at least 48 h to avoid strenuous exercises. All participants were instructed not to eat or drink fluids, other than water, for at least 12 h prior to the experiment, which was performed between 8:00 and 10:00 am. In addition, we ensured that the participants did not consume any dietary sources of nitrate/nitrite or NOS promoters over the 12 h before the test in either group, as the production of NO and nitrate/nitrite can be affected by diet. Thus, we ruled out both the acute effects of the most recent bout of exercise and oral sources of NOx. After the collection of blood samples, they were immediately centrifuged (1500× *g* for 15 min at 4 °C), and stored at −80 °C until measurement. During the experiment, room temperature was maintained at 24 ± 1 °C. The two experiments were performed at a minimum interval of seven days. The participants were instructed to continue their normal activities of daily living and to consume their usual diet throughout the experimental period.

### 2.3. Preparation of Wild Watermelon-Extracted Juice

Wild watermelon-extracted juice was specifically manufactured for this research using the following steps. Wild watermelons (Citrullus lanatus var. citroides) were cultivated in an open field during the summer in Nara Prefecture, Japan. Harvested fruits containing seeds and pericarps were crushed and squeezed using a mixer. The squeezed juice was filtered using an 80 mesh and sterilized at 90 °C for 30 min. The juice (90 g) was adjusted to pH 3 with citric acid, flavored with 50 µL of lemon fragrance oil, and filled up to 100 g with distilled water. In contrast, the placebo was prepared as a drink at pH 3 and consisted of distilled water, citric acid, and lemon fragrance oil.

### 2.4. Measurements of Arterial Stiffness, Blood Pressures, and Heart Rate

Subjects were rested in the supine position for 15 min, and the PWV, BP, and HR of subjects were simultaneously measured using a vascular testing device (form PWV/ABI; Omron Colin, Kyoto, Japan). PWVs are the in vivo gold-standard tool of arterial stiffness assessment [16] and are calculated as the distance between two arterial measuring sites divided by the transit time. A more detailed description of the method for PWVs was included in our previous study [17]. The following PWVs were assessed: brachial-ankle PWV (baPWV), an index of systemic arterial stiffness; carotid-femoral PWV (cfPWV), an index of central arterial stiffness; and femoral-ankle PWV (faPWV), an index of peripheral arterial stiffness.

### 2.5. Measurements of Hemodynamics in Carotid, Brachial, and Posterior Tibial Arteries

Measurements of the carotid, brachial, and posterior tibial artery diameters and mean blood velocities were taken from the arteries on the right side, behind the malleolus medialis of the tibia, using LOGIQ, vivid-q, and S6 ultrasound systems (GE Healthcare, Chicago, IL, USA). A more detailed description of the method used for blood flows was included in our previous study [18].

### 2.6. NOx Levels in Plsama

NOx levels in plasma were measured with Griess assay (R&D Systems, Minneapolis, MN, USA). All samples were assayed in duplicate. Optical density at 540 nm was measured using an xMark microplate spectrophotometer (Bio-Rad Laboratories, Hercules, CA, USA). The sample concentrations were estimated using a linear fit of the log–log plot of the standard curve.

### 2.7. Glucose Levels in Plsama

Glucose levels in plasma were measured with colorimetric assay (Dojindo, Kumamoto, Japan). All samples were assayed in duplicate. Optical density at 450 nm was measured using an xMark microplate spectrophotometer (Bio-Rad Laboratories, Hercules, CA, USA). The sample concentrations were estimated using a linear fit of the log–log plot of the standard curve.

### 2.8. Estradiol Levels in Serum

Estradiol levels in serum were measured with enzyme-linked immunosorbent assay (ELISA; Cayman Chemical, Ann Arbor, Michigan, USA). All samples were assayed in duplicate. Optical density at 414 nm was measured using an xMark microplate spectrophotometer (Bio-Rad Laboratories, Hercules, CA, USA). The sample concentrations were estimated using a linear fit of the log–log plot of the standard curve.

### 2.9. Metabolic Parameters in Serum

Fasting levels of total cholesterol, high-density lipoprotein (HDL) cholesterol, and triglycerides in serum were measured with standard enzymatic techniques. The fasting levels of alkaline phosphatase (ALP), aspartate transaminase (AST), alanine aminotransferase (ALT), and gamma-glutamyl transpeptidase (γ-GTP) in serum were measured as indices of liver function. The fasting levels of creatinine in serum were measured as an index of renal function.

### 2.10. Statistical Analysis

All values were expressed as mean ± standard error. Unpaired Student’s *t*-tests were used to compare any parameters at baseline between the placebo and watermelon intake groups. Percent changes in any parameter were examined using a two-way repeated-measures ANOVA followed by Fisher’s post hoc test, which was applied when the measurement was significantly different. *p* < 0.05 was considered as statistically significant. Using StatView software (version 5.0; SAS Institute, Minato-ku, Tokyo, Japan), all statistical analyses were performed. Based on our previous observations [19,20], the sample size was calculated to be 12 participants using G*Power at an 80% statistical power and an α of 5%.

## 3. Results

### 3.1. SBP, DBP, baPWV, cfPWV, faPWV, and HR

**Figure 1:**
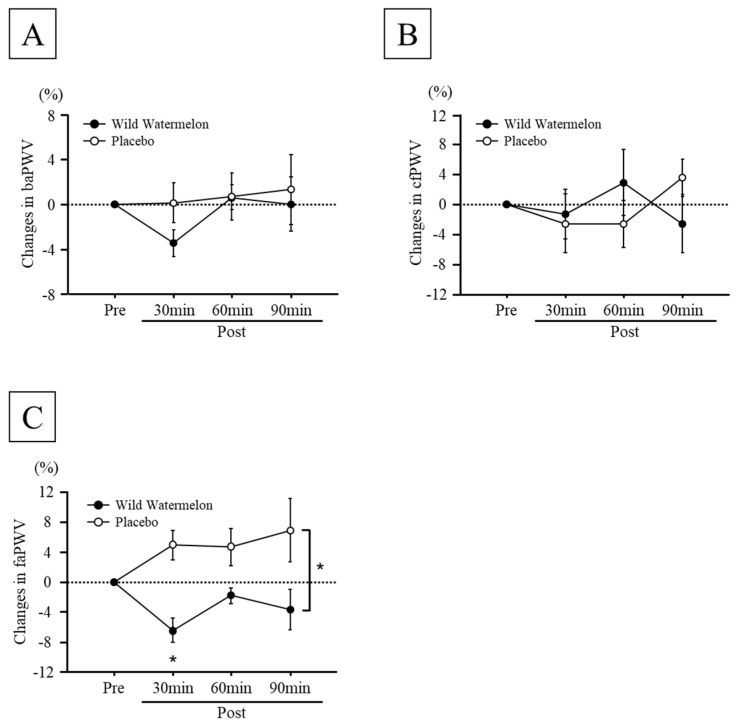
Time course changes in brachial-ankle pulse wave velocity (baPWV) (**A**), carotid-femoral PWV (cfPWV) (**B**), and femoral-ankle PWV (faPWV) (**C**) between wild watermelon and placebo groups. Data are expressed as mean ± standard error. * *p* < 0.05, vs. placebo group.

There were no significant differences in the values of SBP, DBP, baPWV, cfPWV, faPWV, and HR before the intake of each beverage between the two groups (Table 1). There were no significant interactions among the changes in SBP, DBP, baPWV, cfPWV, and HR after beverage intake between the two groups (Figure 1A,B, Table 2). However, a significant interaction in the percentage changes in faPWV was observed (interaction effects: *p* < 0.05, main effects of groups: *p* < 0.0001, main effects of time: *p* = 0.6556, Figure 1). A significant reduction in changes in faPWV was observed in the wild watermelon group at post-30, -60, and -90 min of beverage intake compared to that in the placebo group (Figure 1C; Post-30, *p* < 0.0005; Post-60, *p* < 0.05; Post-90, *p* < 0.05). The changes in faPWV reduced after 30 min of wild watermelon intake (Figure 1C; *p* = 0.0081), but did not change significantly after 60 and 90 min. However, no significant change in faPWV was observed after placebo administration (Figure 1C).

### 3.2. Blood Flows in Carotid, Brachial, and Posterior Tibial Arteries

**Figure 2:**
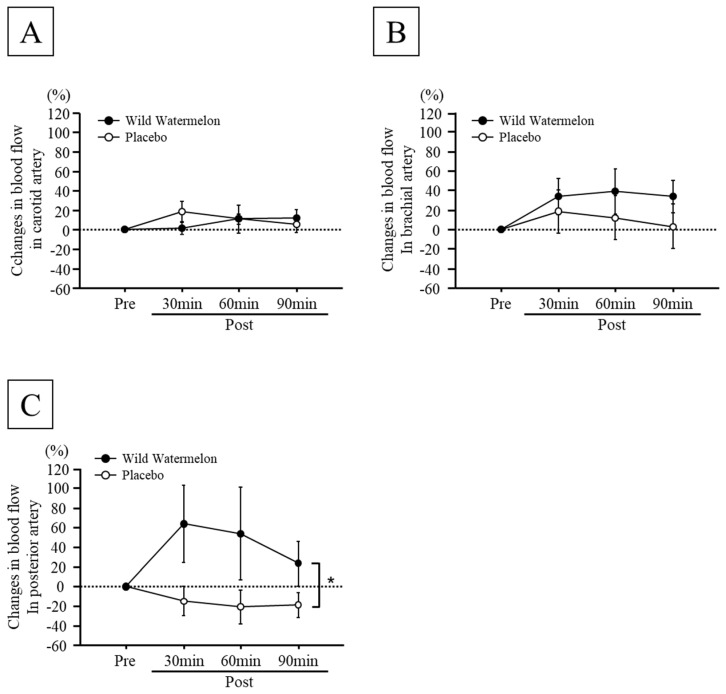
Time course changes in blood flow in carotid artery (**A**), brachial artery (**B**), and posterior artery (**C**) between wild watermelon and placebo groups. Data are expressed as mean ± standard error. * *p* < 0.05, vs. placebo group.

No significant difference was observed in the blood flow in the carotid, brachial, and posterior tibial arteries before intake of each beverage between the two groups (Table 1). Changes in blood flow in the carotid and brachial arteries did not differ between the two groups after each beverage intake (Figure 2A,B). Significant main effects of group on the percentage changes in blood flow in the posterior tibial artery were observed (interaction effects: *p* = 0.3686, main effects of group: *p* < 0.01, main effects of time: *p* = 0.7256, Figure 2C). Changes in blood flow in the posterior tibial artery in the wild watermelon group were significantly increased compared to those in the placebo group (Figure 2C).

### 3.3. Plasma NOx Levels

**Figure 3:**
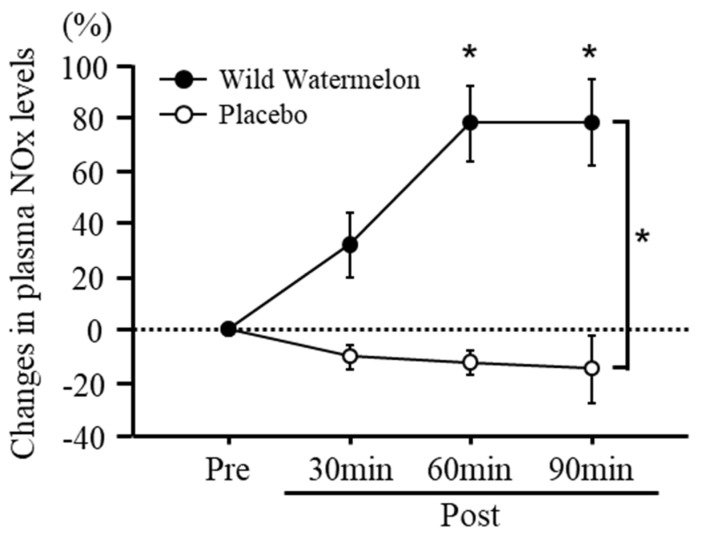
Time course changes in plasma nitrate/nitrite (NOx) levels between wild watermelon and placebo groups. Data are expressed as mean ± standard error. * *p* < 0.05, vs. placebo group.

There was no significant difference in plasma NOx levels before the intake of each beverage (Table 1). A significant interaction in the changes in plasma NOx levels was observed between the two groups (interaction effects, *p* < 0.0001; main effects of group, *p* < 0.0001; main effects of time, *p* < 0.005; Figure 3). A significant increase in plasma NOx levels in the wild watermelon group was observed at post-30, -60, and -90 min of beverage intake compared to that in the placebo group (Figure 3; Post-30, *p* < 0.005; Post-60, *p* < 0.0001; Post-90, *p* < 0.0005). Changes in plasma NOx levels were significantly increased after 60 and 90 min of watermelon intake (Figure 3; 60 min, *p* < 0.0001; 90 min, *p* < 0.0001), but did not significantly change after 30 min. However, no significant change in plasma NOx levels was observed after 30, 60, and 90 min of placebo intake compared to that before placebo intake (Figure 3).

### 3.4. Serum Metabolic Parameters

The circulating glucose, total cholesterol, HDL cholesterol, triglyceride, ALP, AST, ALT, γ-GTP, or creatinine levels did not significantly change before the intake of any beverage between the two groups (Table 1). There were no significant interactions among changes in circulating glucose, total cholesterol, HDL cholesterol, triglyceride, ALP, AST, ALT, γ-GTP, or creatinine levels after each beverage intake between the two groups (Table 2).

## 4. Discussion

The main findings of our study were that wild watermelon-extracted juice increased plasma NOx levels and blood flow in the posterior tibial arteries, and decreased faPWV. However, wild watermelon-extracted juice did not significantly affect the HR, central or systemic arterial stiffness, or blood flow. These results imply that acute ingestion of wild watermelon-extracted juice may effectively accelerate peripheral circulation, although it does not affect central and systemic circulation. This is the first study to demonstrate that acute ingestion of wild watermelon-extracted juice affects peripheral (lower limb) arterial stiffness concomitant with an increase in NO bioavailability.

Wild watermelon is used as a dietary source of water and its seeds are known to contain various essential amino acids [21]. Wild watermelon has high citrulline content, which protects the plant from stress in its native environment [22,23,24]. Citrulline is one of the most potent hydroxyl radical scavengers [25]. Citrulline, a byproduct of the NOS reaction, can be recycled into arginine by argininosuccinate synthetase and argininosuccinate lyase during the citrulline-NO cycle [26]. There have been several reports on the benefits of L-citrulline supplementation on NO bioavailability. A previous study showed that acute ingestion of L-citrulline increased NO bioavailability in healthy young adults [27]. Furthermore, short- and long-term L-citrulline supplementation has been reported to efficiently increase circulating NOx levels [7,13]. Therefore, citrulline, which is present in the juice of wild watermelon extract, may strongly contribute to the increase in NO bioavailability.

The wild watermelon-extracted beverage used in this study is rich in citrulline. In a previous study, an acute intake of L-citrulline supplementation in young healthy adults increased NO bioavailability [27]. Additionally, citrulline malate supplementation increased flow-mediated dilation in the brachial artery, which is an index of arterial endothelial function, by 34% in young male and female adults [28]. Therefore, citrulline extracted from wild watermelon could improve arterial stiffness along with an increase in NO production, although the synergetic effects of citrulline and some nutrients on vascular function must be considered. The juice used in this study contained some nutrients, including lycopene, L-glutamine, malic acid, asparagine, β-carotene, and glutathione. Lycopene injection in the culture medium of human umbilical vein endothelial cell increases NOx concentrations [29]. L-glutamine stimulates blood flow and fluidity by generating NO [30]. Malate and aspartate increase NOS activity in the renal cortex and medulla, resulting in attenuated hypertension in Dahl salt-sensitive hypertensive rats [31]. Additionally, β-carotene injection increased endothelial NOS mRNA levels in cultured human endothelial cells [32]. Furthermore, a combination of L-citrulline and glutathione intake in healthy rats significantly increased plasma NOx levels compared to L-citrulline alone [33]. Thus, these nutrients may synergistically or additively accelerate an increase in NO bioavailability, leading to a reduction in peripheral arterial stiffness. Although our study could not identify any nutrients that affected the vascular function, further studies are required to elucidate them.

In this study, acute ingestion of wild watermelon-extracted juice reduced peripheral arterial stiffness, but not central and systemic arterial stiffness. The heart is primarily responsible for central circulation, and HR is determined by the intrinsic properties of the sinoatrial node and is modulated by the autonomic nervous system [34,35]. In addition, peripheral circulation was affected by acute ingestion of wild watermelon-extracted juice, whereas HR and blood flow in the carotid artery remained unaffected. Peripheral and central arteries have different functions and structures. For instance, it has been reported that the backward pressure wave in the peripheral artery is greater than that in the aorta [36]. Additionally, the vessel wall of the human aorta primarily consists of elastin and collagen fibers and a small number of smooth muscle cells, whereas the tunica media of a muscle-type peripheral artery predominantly consists of smooth muscle cells with scattered elastic membranes and few collagen fibers [37]. These differences in function and structure between the peripheral and central arteries may have affected the data in this study. In fact, a previous study showed that vasodilatory drugs induced preferential muscular arterial dilation, leading to a greater decrease in peripheral (arm and leg) PWV than in central (aortic) PWV [38]. Furthermore, although PWV is affected by both functional and structural changes in the artery walls [39], acute nutritional effects changing the structural artery wall is unlikely. Therefore, the acute ingestion of wild watermelon-extracted juice may preferentially decrease peripheral arterial stiffness, particularly arterial function. A systematic review estimated that peripheral artery disease (PAD) is present in approximately 6% of adults worldwide [40]. Furthermore, PAD is associated with leg pain, walking impairment, and a high risk of major adverse cardiovascular events including amputation and death [41]. Therefore, this study indicates that the intake of wild watermelon-extracted juice may be useful for maintaining an ideal peripheral artery.

A previous study showed that chronic watermelon supplementation increased levels of NOx in plasma concomitant with higher levels of L-arginine and L-citrulline in plasma in healthy young adults [7]. Additionally, chronic intake of watermelon juice for two weeks in healthy young adults improved the area under the curve (AUC) of arterial endothelial dysfunction and the AUC of deteriorated blood flow during experimentally induced acute hyperglycemia [42]. Furthermore, chronic intake of watermelon supplementation for six weeks decreased baPWV [43]. However, the mechanisms underlying this decrease in arterial stiffness remain unclear. The acute stimulatory effect of wild watermelon NO production can lead to chronic accumulation of NO. This study revealed the effects of the acute ingestion of wild watermelon-extracted juice on NO production. Thus, an increase in circulating NOx levels induced by the acute ingestion of wild watermelon-extracted juice might contribute to an increase in circulating NOx levels at basal levels by chronic ingestion.

The acute intake of several bioactive-rich foods positively affects vascular function. For example, eating blueberries acutely improves peripheral arterial stiffness and endothelial function in healthy young men [19,44]. Additionally, the acute consumption of dragon fruit improves endothelial function and arterial stiffness in healthy young men and women [45]. Although several bioactive-rich foods, such as those that affect vascular function, have been reported, the data in this study indicated that wild watermelon, along with the fruit, positively affected vascular function.

This study has several limitations. First, we should consider the menstrual cycle on each measurement date and include young men as well. Second, future studies are required to determine whether or not the wild watermelon-extracted juice affects peripheral arterial function in patients with PAD. Third, we did not assess the dietary habits of the study participants. Fourth, this pilot study had a small sample size. Therefore, further studies are required to confirm the effects of wild watermelon on vascular function, using a large sample of men and patients with PAD.

## 5. Conclusions

In this study, we found that acute ingestion of wild watermelon-extracted juice increased lower-limb blood flow and NO bioavailability, and decreased lower-limb arterial stiffness in healthy women. This finding provides a basis for considering the intake of wild watermelon-extracted juice as a therapeutic strategy for PAD.

## Figures and Tables

**Table 1:** Comparison of characteristics in placebo and watermelon intake groups.

	Placebo	Watermelon	Unpaired *t*-Test
HR, bpm	55.2 ± 2.1	53.2 ± 2.2	0.5100
SBP, mmHg	103.6 ± 1.9	100.5 ± 2.2	0.2884
DBP, mmHg	59.3 ± 1.9	57.5 ± 1.7	0.4658
baPWV, cm/s	997.0 ± 26.4	990.0 ± 22.9	0.8433
cfPWV, cm/s	682.5 ± 10.8	671.4 ± 29.0	0.7235
faPWV, cm/s	847.3 ± 27.5	871.3 ± 30.2	0.5624
BF in carotid artery, mL/min	229.5 ± 19.8	239.6 ± 16.5	0.6962
BF in brachial artery, mL/min	18.8 ± 3.7	16.8 ± 2.0	0.6368
BF in posterior artery, mL/min	9.3 ± 3.5	6.7 ± 2.4	0.5591
Plasma NOx levels, μmol/L	62.0 ± 10.5	63.9 ± 10.8	0.8971
Plasma glucose levels, mmol/L	4.6 ± 0.1	4.6 ± 0.1	0.7953
Serum estradiol levels, pg/mL	152.8 ± 29.6	146.1 ± 21.8	0.8565
Serum HDL cholesterol levels, mg/dL	60.7 ± 3.8	61.9 ± 3.6	0.8121
Serum total cholesterol levels, mg/dL	156.1 ± 4.7	159.1 ± 3.6	0.6168
Serum triglyceride levels, mg/dL	51.2 ± 6.2	47.1 ± 6.6	0.6550
Serum ALP levels, U/L	57.3 ± 4.3	57.1 ± 3.2	0.9756
Serum AST levels, U/L	15.6 ± 1.0	15.5 ± 1.1	0.9557
Serum ALT levels, U/L	9.9 ± 1.6	9.7 ± 1.3	0.9041
Serum γ-GTP levels, U/L	13.3 ± 0.9	13.2 ± 0.9	0.9482
Serum creatinine levels, mg/dL	0.6 ± 0.0	0.6 ± 0.0	0.8789
eGFR, mL/min/1.73 m^2^	101.0 ± 4.2	100.0 ± 4.2	0.8656

HR: heart rate, SBP: systolic blood pressure, DBP: diastolic blood pressure, baPWV: brachial-ankle pulse wave velocity, cfPWV: carotid-femoral pulse wave velocity, faPWV: femoral-ankle pulse wave velocity, BF: blood flow, NOx: nitrate/nitrite, HDL: high-density lipoprotein, ALP: alkaline phosphatase, AST: aspartate aminotransferase, ALT: alanine aminotransferase, GTP: glutamyl transpeptidase, eGFR: estimated glomerular filtration rate. Values are means and SE.

**Table 2:** Percent changes in hemodynamics and metabolic parameters at pre, post-30min, post-60min, and post-90min.

		Post (Placebo Intake)				Post (Wild Watermelon Intake)			Two-Way ANOVA
	ΔPre	Δ30	Δ60	Δ90	ΔPre	Δ30	Δ60	Δ90	
HR, %	0 ± 0	−1.7 ± 1.6	1.1 ± 1.9	−0.4 ± 1.4	0 ± 0	2.3 ± 1.1	3.4 ± 1.6	2.4 ± 1.1	0.4485
SBP, %	0 ± 0	0.3 ± 1.4	−0.1 ± 1.2	2.9 ± 1.4	0 ± 0	−0.2 ± 1.1	2.5 ± 1.1	3.0 ± 1.4	0.5124
DBP, %	0 ± 0	1.1 ± 1.8	−0.5 ± 2.6	3.0 ± 2.7	0 ± 0	−0.8 ± 2.1	1.9 ± 2.4	4.2 ± 2.3	0.7580
Glucose, %	0 ± 0	2.0 ± 1.4	0.4 ± 1.6	1.1 ± 1.3	0 ± 0	2.0 ± 1.2	2.4 ± 1.5	1.6 ± 1.5	0.8234
Estradiol, %	0 ± 0	−1.1 ± 7.4	−8.8 ± 7.0	−12.0 ± 5.8	0 ± 0	−14.3 ± 5.7	−13.0 ± 3.9	−25.4 ± 6.3	0.5007
HDL-C, %	0 ± 0	−3.8 ± 1.4	−2.3 ± 1.0	−1.6 ± 1.7	0 ± 0	−1.7 ± 1.2	−1.4 ± 1.1	−2.5 ± 1.1	0.5731
Total-C, %	0 ± 0	−4.6 ± 1.4	−3.1 ± 1.0	−2.0 ± 1.1	0 ± 0	−2.2 ± 0.9	−1.7 ± 1.0	−2.2 ± 0.8	0.4609
TG, %	0 ± 0	−1.0 ± 2.4	−3.4 ± 1.5	−4.5 ± 2.4	0 ± 0	1.0 ± 2.1	3.4 ± 2.4	2.9 ± 2.6	0.1758
ALP, %	0 ± 0	−5.7 ± 1.8	−2.2 ± 1.2	−2.8 ± 1.1	0 ± 0	−5.7 ± 1.8	−2.2 ± 1.2	−2.8 ± 1.1	0.2180
AST, %	0 ± 0	−8.1 ± 3.7	−4.7 ± 2.1	−1.8 ± 2.7	0 ± 0	−4.4 ± 2.2	−2.1 ± 1.9	−1.6 ± 3.1	0.8144
ALT, %	0 ± 0	−8.5 ± 3.3	−4.9 ± 3.0	1.2 ± 4.0	0 ± 0	−7.4 ± 2.6	−1.2 ± 2.9	−2.8 ± 2.7	0.5636
γ-GTP, %	0 ± 0	−2.2 ± 1.7	−0.6 ± 1.2	−3.1 ± 1.5	0 ± 0	−2.4 ± 2.1	−3.5 ± 1.8	−2.1 ± 2.4	0.6443
Creatinine, %	0 ± 0	−4.7 ± 1.4	−5.5 ± 0.7	−6.7 ± 1.7	0 ± 0	−2.3 ± 1.5	−5.1 ± 1.3	−4.5 ± 1.5	0.6889
eGFR, %	0 ± 0	5.7 ± 1.8	6.5 ± 0.9	8.3 ± 2.3	0 ± 0	2.9 ± 1.7	6.1 ± 1.7	5.5 ± 1.8	0.6757

HR: heart rate, SBP: systolic blood pressure, DBP: diastolic blood pressure, HDL-C: high-density lipoprotein cholesterol, Total-C: total cholesterol, TG: triglyceride, ALP: alkaline phosphatase, AST: aspartate aminotransferase, ALT: alanine aminotransferase, GTP: glutamyl transpeptidase, eGFR: estimated glomerular filtration rate. Δ: Percent changes from Pre in each parameter, Values are means and SE.

## Data Availability

The data presented in this study are available upon request from the corresponding author.
